# Consequences of point mutations in melanoma-associated antigen 4 (MAGE-A4) protein: Insights from structural and biophysical studies

**DOI:** 10.1038/srep25182

**Published:** 2016-04-28

**Authors:** Yoshio Hagiwara, Lina Sieverling, Farina Hanif, Jensy Anton, Eleanor R. Dickinson, Tam T. T. Bui, Antonina Andreeva, Perdita E. Barran, Ernesto Cota, Penka V. Nikolova

**Affiliations:** 1 King’s College London, Faculty of Life Sciences & Medicine, Institute of Pharmaceutical Science, Franklin-Wilkins Building, 150 Stamford St, London, SE1 9NH, UK; 2 Michael Barber Centre for Collaborative Mass Spectrometry, Manchester Institute of Biotechnology, The University of Manchester, 131 Princess Street, Manchester, M1 7DN, UK; 3 Biomolecular Spectroscopy Centre, King’s College London, The Wolfson Wing, Hodgkin Building, London SE1 1UL; 4 MRC-LMB, Francis Crick Avenue, Cambridge, CB2 0QH; 5 Imperial College London, Faculty of Natural Sciences, London, SW7 2AZ.

## Abstract

The Melanoma-Associated Antigen A4 (MAGE-A4) protein is a target for cancer therapy. The function of this protein is not well understood. We report the first comprehensive study on key cancer-associated MAGE-A4 mutations and provide analysis on the consequences of these mutations on the structure, folding and stability of the protein. Based on Nuclear Magnetic Resonance and Circular Dichroism, these mutations had no significant effects on the structure and the folding of the protein. Some mutations affected the thermal stability of the protein remarkably. Native mass spectrometry of wild-type MAGE-A4 showed a broad charge state distribution suggestive of a structurally dynamic protein. Significant intensity was found in relatively low charge states, indicative of a predominantly globular form and some population in more extended states. The latter is supported by Ion Mobility measurements. The MAGE-A4 mutants exhibited similar features. These novel molecular insights shed further light on better understanding of these proteins, which are implicated in a wide range of human cancers.

Cancer/Testis (CT) antigens are a large family of proteins typically expressed in germ cells. CT antigens can also be overexpressed in an aberrant manner in various types of tumors such as melanoma, sarcoma, lung cancer, prostate cancer, breast cancer, ovarian cancer and a range of various other cancers^1^,^2^,^3^,^4^,^5^.

The first discovered CT antigen family member was melanoma-associated antigen A1 (MAGE-A1), which was identified as an antigen recognised by cytotoxic T lymphocytes in melanoma patients^6^, and was successfully cloned later^7^,^8^. Since then, extensive chromosomal sequencing identified more than 50 genes of the MAGE family^9^,^10^,^11^,^12^,^13^,^14^,^15^,^16^,^17^,^18^. Based on their expression pattern, the MAGE family can be divided into two subfamilies: type-I and type-II^19^. While the expression of type-I subfamily is restricted to germ line and cancer cells, type-II subfamily is expressed in normal somatic tissues. Type-I MAGEs can be subdivided into MAGE-A, -B and -C groups. MAGE-A contains 12 genes (MAGE-A1 to -A12) where MAGE-A7 is a pseudogene^9^. MAGE proteins consist of nearly 100 amino acid residues long N-terminal region, followed by two tandem winged helix domains with each wing termed as WH-A and WH-B, respectively^20^. The N-terminal is rich in disorder promoting residues such as Ser, Pro, Glu and Arg, and thus this region is predicted to be disordered. The C-terminal region that spans over the two winged helix domains is highly conserved amongst type I and type II MAGEs and is known as MAGE Homology Domain (MHD). The crystal structure of MHD has been determined in a free state (MAGE-A4, PDB ID: 2AW0; MAGE-A3, PDB ID: 4V0P) and in complex with NSE1 (MAGE-G1, PDB ID: 3NW0)^20^.

The biological functions of MAGE proteins remain poorly understood. However, many reports correlate over-expression of type-I MAGEs with cancer malignance, tumor growth and poor patient prognosis. For example, MAGE-A2 was reported to promote tumor growth in normal oral keratinocytes and inhibits cell cycle arrest through down-regulation of p53 targets^21^. MAGE-A4 was also shown to abrogate p53-dependent growth arrest and apoptosis in normal oral keratinocyte^22^. The mechanism(s) of action of MAGE-A proteins that can lead to tumor growth have not yet been fully elucidated at a molecular level. However, MAGE-A2 was reported to interact with the DNA binding domain of p53 and to suppress p53 trans-activation by recruiting histone deacetylase (HDAC)^23^ or by inhibiting the interaction between p53 and DNA through steric hindrance^24^. Most recently, MAGE-A antigens were identified as being causal contributors in the development of tamoxifen-resistant breast cancers^25^. Specifically, MAGE-A2 protein was reported to localize to the nucleus and to form complexes with p53 and ER alpha, resulting in repression of the p53 pathway while increasing the ER dependent signalling. This report showed that in tamoxifen-treated breast cancer patients, there was a significant link between MAGE expression and reduced overall survival. In addition, various MAGE proteins such as MAGE-A2, -A3 and -A6, have been reported to interact with E3 ubiquitin ligase such as TRIM28, enhancing their ubiquitin ligase activity and reducing p53 protein level through a proteasome-dependent pathway^20^.

In contrast, tumor suppressor activity was also reported for MAGE-A4. MAGE-A4 was found to interact with the liver oncoprotein gankyrin, suppressing its tumorigenic activity^26^. A truncated form of MAGE-A4 (the C-terminal 107 amino acids) was reported to induce apoptosis by interacting with POZ domain/zinc finger transcription factor Miz-1^27^.

To date, there are 104 MAGE-A4 DNA mutations documented in the Catalogue of Somatic Mutations in Cancer (COSMIC) database (http://cancer.sanger.ac.uk/cancergenome/projects/cosmic/). Among these mutations, 76 are missense mutations, 52 of which are located within the MHD. In addition, Caballero *et al*.^28^ identified ten missense mutations in melanoma patients and two missense mutations in tumor samples (breast cancer cell line and glioma)^28^.

In this study, we have selected nine MAGE-A4 mutations to investigate the effects of these mutations on the structure, folding and stability of MAGE-A4, all of which are reported by Caballero *et al*.^28^. Eight of these mutations are located within the MHD (E138K, P149S, G153D, E221K, E224K, E242K, P267S and R269C) and one near the C-terminus (G316R). The overall criteria for the selection of these mutations was that they mapped within the MHD of MAGE-A4, which is the only domain that had known X-ray structure. Our results reveal that although the mutations have marginal effects on the structure of MAGE-A4, some of them can affect significantly the thermal stability of the protein. In addition, both WT and mutant MAGE-A4 contain a large disordered or unfolded region as well as a structured hydrophobic core. Native mass-spectrometry (MS) shows that all of the MAGE-A4 proteins are present primarily in a monomeric form with a low abundant fraction of dimeric species. Ion mobility mass spectrometry (IM-MS) shows the WT MAGE-A4 to present in multiple conformers, whilst mutant MAGE-A4 proteins show significant compaction in the gas-phase. This study provides novel information for one of the MAGE-A proteins, namely, MAGE-A4 with regard to its biophysical properties. We have performed structure-based homology modelling, mass-spectrometry (MS), circular dichroism (CD) and nuclear magnetic resonance (NMR) to provide comprehensive analyses of the MAGE-A4 protein and its key cancer associated mutants. The finding could be exploited for cancer-based drug design and/or to inform further cancer therapies.

## Results

### Assessing the folding of the MAGE-A4 proteins using Far-UV CD at different temperatures

**Figure 1:**
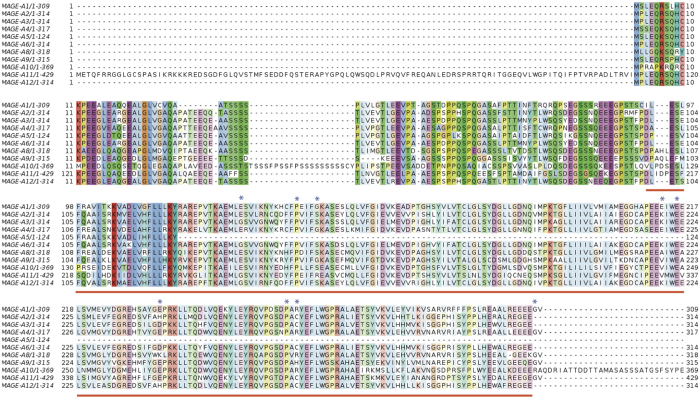
Multiple sequence alignment of MAGE-A proteins. Sequences are colored according to the Clustal coloring scheme. The MHD region is underlined in red. Residues that are mutated in this study are indicated with a star.

**Figure 2:**
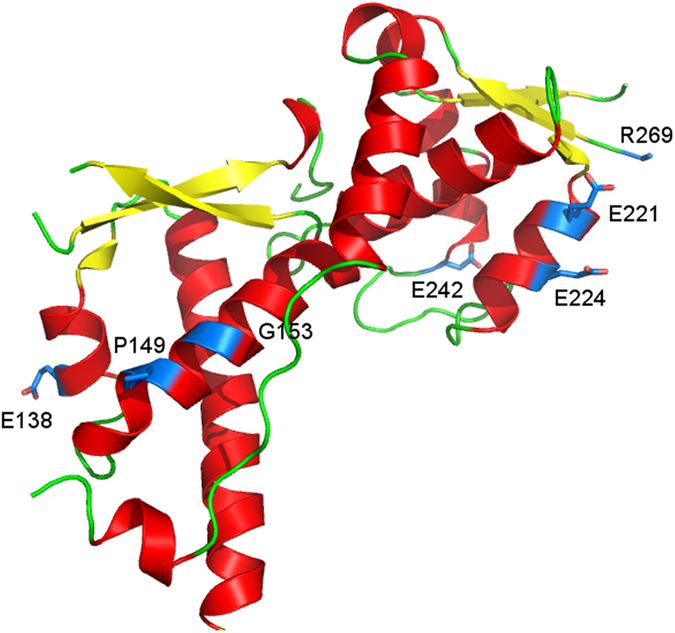
Crystal structure of MAGE-A4 MHD (PDB ID: 2WA0). The structure depicts the secondary structure helices in red and strands in yellow. The mutated residues that have been investigated in this study are shown in blue. Note that P267 and G316 are not visible in the structure. The figure was produced using Pymol.

**Figure 3:**
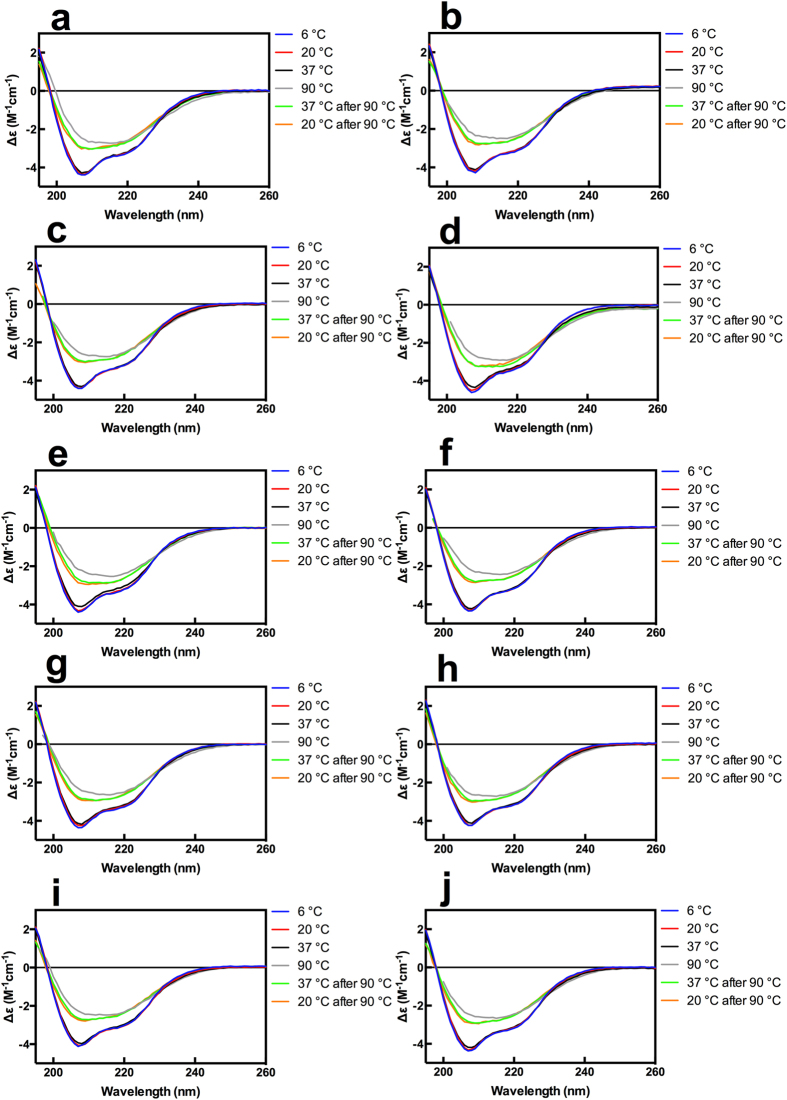
Far-UV CD spectra of WT and mutant MAGE-A4 proteins. Spectra in the region of 260–195 nm were obtained at 6 °C, 20 °C, 37 °C, 90 °C, 37 °C after heating to 90 °C, and at 20 °C after heating to 90 °C. (**a**) WT, (**b**) E138K, (**c**) P149S, (**d**) G153D, (**e**) E221K, (**f**) E224K, (**g**) E242K, (**h**) P267S, (**i**) R269C and (**j**) G316R.

In order to assess the folding state and secondary structure of MAGE-A4 and examine the effects of mutations on them, the proteins were subjected to CD in the Far-UV region (260–195 nm). The locations of these selected mutations in the amino acid sequence and the crystal structure of MAGE-A4 MHD are shown in Figs 1 and 2, respectively. The spectra were recorded at 6, 20, 37 and 90 °C, followed by cooling down to 37 °C and 20 °C respectively. The results are presented in Fig. 3. The CD spectra of each protein at 6, 20 and 37 °C were essentially identical, indicating that the WT and mutant MAGE-A4 proteins maintain their native folded state at physiological temperature. In addition, no significant difference between the WT and the mutants was observed in the spectra at each temperature, suggesting little effects of these mutations on the secondary structure. When the temperature was raised to 90 °C, all of the proteins were thermally denatured. When the temperature was cooled down to 37 and 20 °C, the spectra still clearly differed from those obtained at 6, 20 and 37 °C, demonstrating that the proteins did not recover their native folded state. This indicates that the thermal unfolding of MAGE-A4 proteins is irreversible. We employed the K2D3 programme (http://k2d3.ogic.ca/)^29^ to estimate the secondary structure elements of WT MAGEA-4 at physiological temperature (*i.e.* 37 °C), and this was estimated to be 29.9% α-helix and 17.1% β-sheet.

### The effects of mutations on the thermal stability of MAGE-A4

**Figure 4:**
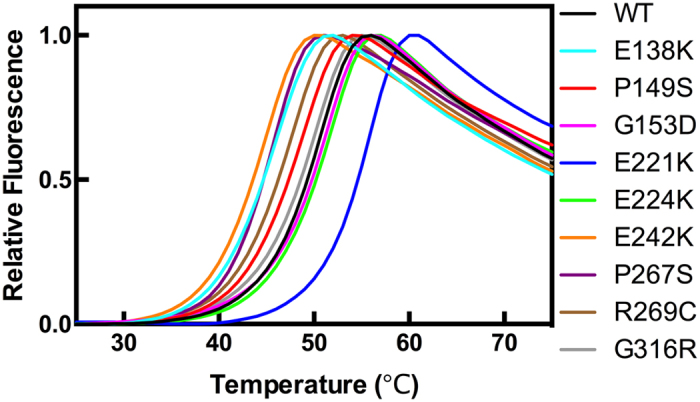
Thermal unfolding curves of MAGE-A4 and its cancer-associated mutants recorded by DSF. The data were recorded using MX3005P machine (Strategene) with the excitation and emission of SYPRO Orange dye set to be 470 nm and 570 nm, respectively. The proteins were diluted to 2 μM in 50 mM Tris buffer containing 150 mM NaCl, 5 mM DTT and 5% glycerol, pH 7.2.

We employed differential scanning fluorimetry (DSF) to determine the apparent melting temperature (*T*_m_) of MAGE-A4 proteins and assess the effects of the mutations on the thermal stability of the protein. The melting curves of MAGE-A4 proteins are shown in Fig. 4. All of the curves have the classic sigmoidal shape showing two different phenomena: unfolding and aggregation. The unfolding process of each protein occurs in the range of 20 °C. The apparent *T*_m_ of WT MAGE-A4 is ~49.3 °C. The least thermally stable protein was E242K with the *T*_m_ ~ 43.6 °C, followed by P267S (*T*_m_ ~ 44.6 °C), E138K (*T*_m_ ~ 44.7 °C), R269C (*T*_m_ ~ 46.2 °C) and P149S (*T*_m_ ~ 47.3 °C). The difference between the *T*_m_ of E242K and that of WT, (Δ*T*_m_), was −5.7 °C, and thus significant. Nearly 10% fraction of E242K mutant unfolds at physiological temperature. The most stable mutant was E221K, showing *T*_m_ ~ 54.6 °C and Δ*T*_m_ of 5.3 °C. The difference in the *T*_m_ between the most stable mutant (E221K) and that of the least stable mutant (E242K) was remarkable (11 °C). Other mutants (G153D, E224K and G316R) showed similar thermal stability to the WT with relatively similar *T*_m_, within ~1 °C difference compared to the WT. These results demonstrate that some mutations can dramatically enhance (such as E221K) or reduce (such as E242K) the thermal stability of the protein. Interestingly, the mutations that showed significant difference in the thermal stability of the proteins (E242K and E221K) affect solvent-exposed residues.

### Molecular modelling

**Figure 5:**
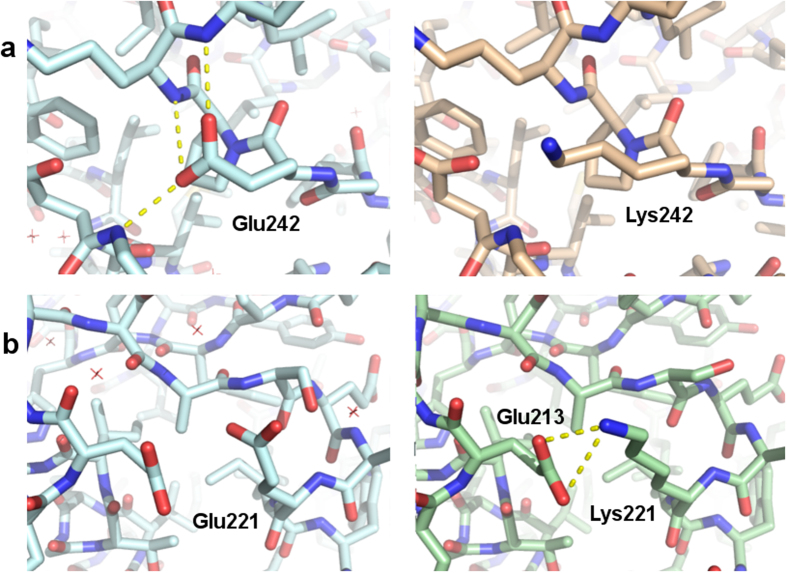
(**a**) Side by side comparison of the structure of wild type MAGE-A4 (PDB ID: 2WA0) and the theoretical model of the E242K mutant. (**b**) Side by side comparison of the structure of wild type MAGE-A4 (PDB ID: 2WA0) and the theoretical model of the E221K mutant.

Using Modeller^30^ we modelled the point mutations in the MAGE-A4 structure in order to understand the structural basis for the observed differences in the thermal stability. The residues Glu242 and Glu221 have the most pronounced effect on the stability. The Glu242 residue is located at the N-cap of helix 3 of the second winged helix domain. It H-bonds to the exposed backbone NH of the residues Arg244 and Lys245 in the first helical turn (Fig. 5a). This residue is fairly conserved amongst MAGE-A4 homologs suggesting the importance of this position. The mutation to Lys could cause a loss of the H-bonding network and this can account for the drastic loss of stability we have observed. The Glu221 residue is located on the second helix of the second winged helix domain. Mutation to Lys could allow the formation of a salt bridge between Lys221 and Glu213 and this can potentially increase the stability of the mutant E221K MAGE-A4 protein (Fig. 5b). The effect of the remaining mutations on the structure is less obvious. The Pro267 and Arg269 residues are located at a loop region that is disordered in the crystal structure. Mutation of both residues may have a destabilizing conformational effect that can vary in magnitude depending on the residue location within the loop. The Pro149 and Gly153 residues are located on the third helix of the first winged helix domain. Both residues are probably important for the stability of the alpha-helix, particularly Pro149, as it is located at the N-terminal turn of the helix and is entropically favoured in this position. A substitution of Gly153 with a residue with side chain and charge such as Glu would be sterically unfavoured as it may clash with the side chain of the residue Ile300. The Glu138 residue is located at helix 2 of the first winged helix domain. Mutation of this residue to Lys may be unfavoured due to charge repulsion as this residue is in close vicinity to Lys142, Lys145 and Arg139.

### 1D proton NMR of MAGE-A4 proteins

**Figure 6:**
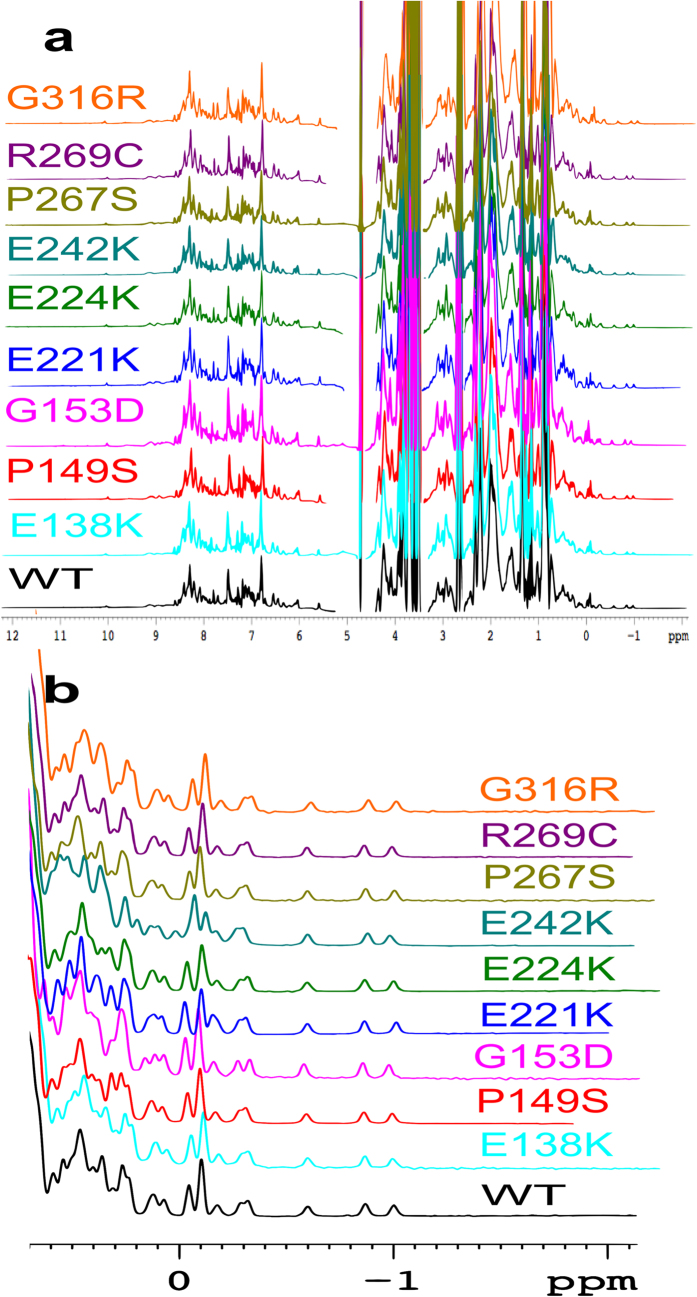
1D Proton NMR Spectra of WT MAGE-A4 and its cancer-associated mutants. (**a**) Whole Spectra. (**b**) Zoomed-in spectra within 0.7 and −2 ppm. Each protein was concentrated to 200 μM in 50 mM phosphate buffer (pH 7.2) containing 50 mM NaCl and 5 mM DTT. The spectra were recorded at 600 MHz, 25 °C.

1D proton NMR was employed to examine the folding and conformation of MAGE-A4 protein and to assess the effects of the mutations in such properties. The spectra of the proteins are shown in Fig. 6a. The differences in the spectra between the WT and mutant proteins are minimal and located within the chemical shift of methyl protons (below 0.8 ppm). The NMR spectra for all of the tested MAGE-A4 proteins showed excellent dispersion of peaks between −1 and 0.8 ppm that are typical of ring-current shifted protons located above or below the plane of aromatic side-chain. This indicates the presence of a structured hydrophobic core. However a large peak at <1 ppm was also observed for each protein, which is indicative of unstructured and disordered regions. These results suggest that the WT and mutant MAGE-A4 proteins contain a folded structure as well as disordered region(s). Figure 6b zooms in the chemical shift region below 0.8 ppm where the difference in the peaks is observed. While the spectra of P267S and R269C overlap completely with that of WT, the spectra of other mutants differ from that of WT in the chemical shift region from 0.7 to −0.2 ppm. These small changes in the positions and patterns of the peaks reflect minor changes in the hydrophobic core caused by such mutations even though these mutations are located on the solvent-exposed surface of the protein. These mutations may somehow change the position or orientation of the planes of aromatic and/or methyl groups, changing the distances and/or angles of the interactions between methyl groups and aromatic planes.

### MS and IM-MS of MAGE-A4

**Figure 7:**
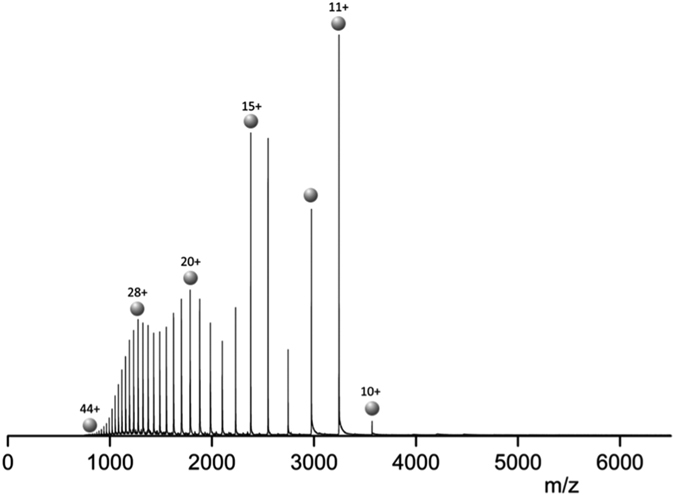
nESI mass spectrum for 20 *μ*M WT MAGE-A4. WT MAGE-A4 sprayed from aqueous solution containing 50 mM Ammonium Acetate at pH 6.8. The protein exhibits a wide, multimodal, monomeric charge state range 10 ≤ *z* ≤ 44. Several charge states are denoted with single spheres along with their respective charge.

We employed MS and drift time IM-MS to probe the structure of WT and mutant MAGE-A4. The mass spectrum for WT MAGE-A4 is shown in Fig. 7. The protein presents in a wide charge state distribution (CSD) 10 ≤ *z* ≤ 44. The most intense charge state is [M+11H]^10+^ although the distribution is multimodal and other intense species include [M+15H]^15+^, [M+20H]^20+^ and [M+28H]^28+^. The CSD was easily malleable, changing significantly with ionic strength and cone potential. As the salt concentration increased the intensity of the higher charge states decreased somewhat, but they were still present over the same range (data not shown). The protein predominantly presents as a monomer of mass 35, 679 (cf. predicted mass of 35,684 Da) with very low abundance peaks assigned to a dimeric form (Supplementary Fig. S1). The wide CSD is typical of an intrinsically disordered protein, with many solvent accessible protonatable sites^31^. The intense [M+11H]^11+^ species indicates WT MAGE-A4 may possess some stable secondary structure, or at the very least a globular form which is built around the solved MHD structure.

**Figure 8:**
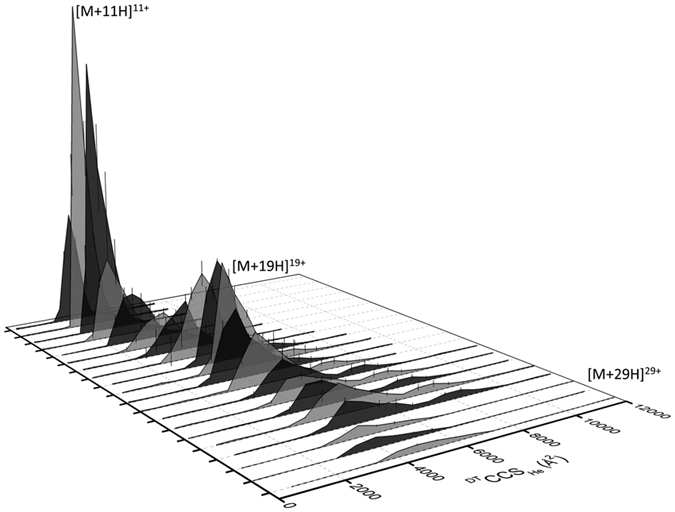
Collision cross section distributions (CCSDs) derived from arrival time distributions (ATDs) for WT MAGE-A4 at drift voltage 35 V. The x-, y- and z-axes represent collision cross section (CCS, Å^2^), intensity directly acquired from IM-MS experiment, and charge state, respectively. Significant charge states have been labelled.

IM-MS reveals that WT MAGE-A4 presents in multiple conformational families (Fig. 8). The low charge states [M+10H]^10+^, [M+11H]^11+^ and [M+12H]^12+^ present in a single conformational family at collision cross section (CCS) ~2400 Å^2^, likely corresponding to a stable compact form of the protein. At intermediate charge states 13 ≤ *z* ≤ 20 MAGE-A4 presents in at least two poorly resolved conformational families centered at ~3200 and 4500 Å^2^. At high charge states 21 ≤ *z* ≤ 26 the protein exhibits very wide collision cross section distributions (CCSDs) with multiple conformational families present ranging from ~3500 to 7500 Å^2^, indicative of a disordered protein with large extended states. The highest charge states 27 ≤ *z* ≤ 29 exhibit a single broad CCSD centered on ~4000 Å^2^, possibly due to gas-phase collapse. Charge states higher than [M+29H]^29+^ were not of sufficient intensity to obtain IM-MS data.

Mutant MAGE-A4 spectra are shown in Supplementary Fig. S2. All mutants present a wide CSD similar to that of WT MAGE-A4. For each case the proteins present as mainly monomer, exhibiting a multimodal distribution with the [M+11H]^11+^ species being the most intense. E138K MAGE-A4 presents in a monomeric charge state range 8 ≤ *z* ≤ 35 with some low intensity low charge dimers. P149S MAGE-A4 presents in charge state range 8 ≤ *z* ≤ 33, with a higher abundance of dimeric species compared with the WT protein. G153D MAGE-A4 presents as a charge state range 7 ≤ *z* ≤ 19, with significantly fewer high charge states compared with the WT protein. As for WT MAGE-A4, G153D MAGE-A4 presents with very low abundance dimeric species. E221K MAGE-A4 presents in charge state range 7 ≤ *z* ≤ 21 with some low intensity low charge dimeric species. E224K presents in charge state range 7 ≤ *z* ≤ 20, again with the [D+15H]^15+^ and [D+17H]^17+^ dimeric species. E242K presents in a charge state range 7 ≤ *z* ≤ 18 with low intensity [D+15H]^15+^ dimer. This charge state range is narrower, and dimer abundance is lower than the WT MAGE-A4 protein. P267S MAGE-A4 presents in a monomeric charge state range 7 ≤ *z* ≤ 22. The dimeric species are low intensity with charge state range 15 ≤ *z* ≤ 39, the highest abundance of dimeric species of WT and mutant MAGE-A4. R269C presents as a monomeric charge state range 7 ≤ *z* ≤ 17, with [D+15H]^15+^ and [D+17H]^17+^ dimers. G316R presents with a monomeric charge state range 7 ≤ *z* ≤ 31 and a dimeric charge state range 15 ≤ *z* ≤ 25.

All mutants present with a narrower charge state range compared with the WT MAGE-A4, with G153D, E242K and R269C displaying a significantly narrower distribution, centered on lower charges suggestive of a more compact form being dominant in solution. MAGE-A4 mutants P149S, P267S and G316R display an increased abundance of dimeric species compared with the WT protein.

We performed IM-MS on the MAGE-A4 mutants which exhibited significant thermal stability differences; the most stable, E221K, and the least stable, E242K MAGE-A4 (Supplementary Fig. S3). E221K MAGE-A4 presents in similar conformational families to the WT protein for low charge states 9 ≤ *z* ≤ 12, with a single conformational family centered at ~2400 Å^2^. At intermediate charge states 13 ≤ *z* ≤ 16 E221K MAGE-A4 exhibits two conformational families centered at ~2600 and ~4000 Å^2^; similar to that of WT MAGE-A4 but with a narrower distribution. The high charge states 17 ≤ *z* ≤ 20 present in much more narrow CCSDs for that observed in the WT protein. We see no highly extended conformational states, instead observing a broad unresolved distribution at ~3500–5000 Å^2^. The E242K MAGE-A4 protein again exhibits conformational differences in comparison with WT MAGE-A4. The trend of a single conformational family at low charge states, with multiple conformational families presenting at charge states 13 ≤ *z* ≤ 23 remains consistent. We again however do not observe the more unfolded states present in the high charge states of WT MAGE-A4, and observe consistently much narrower CCSDs than both WT and E221K MAGE-A4. Several charge states are directly compared for the WT, E221K and E242K MAGE-A4 proteins (Supplementary Fig. S4), highlighting the similar CCSDs for the compact, low charge states but significant differences in the CCSD width at both intermediate and especially high charge states for the two mutant MAGE-A4 proteins. The mutations which impact on thermal stability, both positively and negatively, appear to also have an effect on the conformation landscape of MAGE-A4. The narrowing of the CCSDs suggests that E221K and E242K MAGE-A4 proteins possess less flexibility than WT MAGE-A4, preferentially presenting in more compact conformational states.

## Discussion

Despite being discovered more than 20 years ago, biological functions of the MAGE family of proteins still remain poorly understood. Most studies were focused on developing anti-cancer immune therapy using the exclusiveness of type-I MAGE expression in cancer cells. For example, MAGE-A3 has been studied as a candidate for developing immune therapy^32^,^33^,^34^. Emerging data highlight their active roles in promoting cancer growth and malignance^35^,^36^,^37^,^38^,^39^. MAGE-A2 has been reported to repress the activity of tumor suppressor protein p53 through recruitment of HDAC or by inhibiting the interaction between p53 and DNA^23^,^24^.

In this study, we have selected MAGE-A4 and nine of its mutants identified in human cancer cells to obtain a first insight into their structural and biophysical properties. We observed that the selected point mutations have little effects on the structural integrity and folding of the protein probably since most of these mutated residues are located on solvent-exposed surface. However some mutations, such as E221K and E242K, had significant effects on the thermal stability. The apparent *T*_m_ of E242K MAGE-A4 was 5.7 °C lower than that of WT protein. A small fraction (*ca*10%) of this MAGE-A4 mutant unfolds at physiological temperature. The decreased thermal stability coupled with unfolding at physiological temperature caused by E242K mutation may reduce the functional activities of MAGE-A4 protein *in vivo*.

Both NMR and native MS showed for the first time that these MAGE-A4 proteins contain a compact folded structure as well as disordered region(s). Most of such disordered regions are expected to be located within the N-terminal 100 residues due to high presence of disorder promoting residues. Interestingly, we observed a very small fraction of dimeric species in the native MS. It has been reported that MAGE-A11 can form dimer^40^. In addition, while this article was under review, a new report emerged suggesting that MAGE-A3 presents predominantly as dimer in solution^41^. It is not known whether such dimeric species possess different functional properties compared to the monomers.

Of note, the percentage of MAGE-A4-positive patient tumors observed in select cancer subtypes has been reported as follows: 47% in ovarian cancers, 19–35% in lung cancers, 22% in colon cancers and 13% in breast cancers^39^. The role of MAGE-A4 in cancers is not fully understood especially at macromolecular level. In fact, there are conflicting reports regarding its role in cancer. It has been reported to have oncogenic properties by inhibiting p53 downstream genes such as *BAX* and *p21*, and thus promoting cell growth in normal oral keratinocytes^22^. However MAGE-A4 was also reported to have tumor suppressive functions as it was shown to promote apoptosis in non-small cell lung cancers^42^. It is not understood how MAGE-A4 exerts such opposing roles. We hypothesize that such observations may arise from different interacting partners of MAGE-A4. As part of its tumor repressing mechanism, MAGE-A4 has been indicated to bind with gankyrin, suppressing its oncogenic activity^26^. However, to date, no direct molecular interactions have been identified for MAGE-A4, which can lead to cancer growth.

As the point mutations selected for this study are located on the solvent exposed surface of MAGE-A4, even though they do not have effects on structural integrity of MHD as observed in this study, they may have a significant impact on the protein-protein interaction(s), which may enhance or repress tumor growth. In addition, conformational changes have been shown for E221K and E242K mutants in IM-MS experiments. These may also have effects on the protein-protein interaction(s), by modifying the conformational flexibility of MAGE-A4. Furthermore, the impact of the mutations on the thermal stability and folding of the protein may have significant effects on the functions of this protein *in vivo*. Further investigation needs to be conducted to identify other proteins that interact with MAGE-A4 and assess the impact of these mutations on such interactions to understand whether these mutations have different functional roles in cancer.

Given the significance of MAGE-A4 in biology and specifically in cancer, it is important to fully understand the functions of this protein at the molecular level in order to exploit it in translational research. Specifically, the mutations may affect the interactions with key binding cell cycle proteins. The latter are subject to further investigations aiming to identify and characterise such binding proteins in the hope to better understand the role of these mutations in cancer.

## Methods

### MAGE-A4 cloning and site-directed mutagenesis

The DNA of WT MAGE-A4 was cloned into pGEX-6p-1 (GE Healthcare) to express GST-tagged MAGEA-4 using primer-1: 5′-AAAAAAGAATTCATGTCTTCTGAGCAGAAGAGTCAG-3′ as a forward primer, primer-2: AAAAAACTCGAGTCAGACTCCCTCTTCCTCTAA-3′ as a reverse primer, and vector pGEX-4T-1 containing MAGE-A4 DNA sequence^43^ as a template. MAGE-A4 mutants were generated using QuickChange^®^ Lightning Site-Directed Mutagenesis Kit (Agilent Technologies).

### Protein expression and purification

Proteins were expressed in *Escherichia coli* C41 (DE3) cells. Single colonies were inoculated into 5 ml LB media (ForMedium) supplemented with 100 μg/ml ampicillin, followed by incubation at 37 °C overnight. 5 ml of the overnight culture was added into 500 ml LB with 100 μg/ml ampicillin, followed by incubation at 37 °C until the OD_600_ reached 0.6. The culture was then allowed to cool down to 21 °C and the protein expression was induced with 1 mM isopropyl β-D-thiogalactoside overnight. The cultures were centrifuged at 11300 × g, 4 °C for 20 min. The cells were collected and lysed using 5 ml/g cell of BugBuster^®^ protein extraction reagent (Novagen) containing 1 tablet of complete^®^ EDTA- free protease inhibitor (Roche), 125 U of Benzonase nuclease (Novagen) and 5 mM dithiothreitol (DTT). The cell lysate was centrifuged at 20000 × g, 4 °C for 40 min. The soluble fraction was collected and the protein was purified using GST GraviTrap columns (GE Healthcare). The GST-tagged protein was incubated with 5 U/mg protein of PreScission protease (GE Healthcare) at 4 °C overnight. The protein was passed again through GST GraviTrap column to remove the GST, followed by further purification using 26/60 Superdex 200 column connected to ÄKTAprime^®^ system equipped with Prime View software (GE Healthcare). The purified protein was concentrated using Amicon Ultra-15 centrifugal filter unit, 10,000 NMWL (Merck Millipore) and stored at −80 °C.

### CD

Simultaneous UV absorption and CD spectra of MAGE4 proteins were acquired on the Chirascan Plus^®^ spectrometer, equipped with a Quantum Northwest TC125 Peltier unit (Applied Photophysics, Leatherhead, UK). The instrument was flushed continuously with pure evaporated nitrogen gas throughout the measurements. Far-UV CD spectra were recorded with a 2.0 nm spectral bandwith, a 1.0 nm step size and a 1.5 s instrument time-per-point. A 0.5 mm Suprasil rectangular cell (Hellma UK Ltd) was employed in the region 260–195 nm. Far-UV CD spectra were recorded at room temperature (20 °C), cooled to 6 °C, then heated to high temperatures (37 °C and 90 °C) and re-cooled to 37 °C and 20 °C. The temperature was measured directly with a thermocouple probe in the sample solution. Protein samples were concentrated to 0.2 mg/ml and were buffer exchanged to 20 mM Tris buffer containing 50 mM NaCl and 1 mM dithioerythritol (DTE), pH 7.2. All CD spectra were buffer baseline subtracted and then corrected for concentration and path length and expressed in terms of Δε (M^−1^cm^−1^) per amino acid residue (MW = 113). Protein secondary structure prediction was performed using K2D3 (http://k2d3.ogic.ca/)^29^.

### DSF

Protein samples were diluted to 2 μM in 50 mM Tris buffer containing 150 mM NaCl, 5 mM DTT, and 5% glycerol. SYPRO^®^ Orange stain (Sigma-Aldrich) was added into the samples to be diluted 500 times. 20 μl of each sample was transferred to 96-well plate, and the plate was sealed with a transparent tape. The samples were run in MX3005P machine (Strategene) connected to MxPro software and the temperature was increased from 25 °C to 95 °C. The stain was excited at 470 nm and the emission at 570 nm was measured. The unfolding curve was generated by normalizing the fluorescence values using equation:


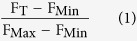


where F_T_ is fluorescence at temperature T, F_Max_ is maximum fluorescence, and F_Min_ is minimum fluorescence.

### Molecular modelling

Models were built with Modeller^30^ using the structure of human MAGEA4 as template (PDB ID: 2WA0).

### 1D proton NMR

Protein samples were concentrated to 200 μM and the buffer was exchanged to 50 mM phosphate sodium buffer containing 50 mM NaCl and 5 mM DTT. 500 μl of each protein sample was mixed with 50 μl of deuterium oxide and transferred to an NMR tube. The proton NMR spectra were recorded using Bruker Avance III 600 at 25 °C, 600 MHz.

### MS

Prior to analysis, samples were thawed and dialyzed using Bio-RAD micro bio-spin chromatography columns (Bio-Rad Laboratories, Inc) into 50 mM Ammonium Acetate and diluted to 20 μM. Protein concentrations were confirmed post dialysis spectrophotometrically (NanoDrop Spectrophotometer ND 1000, Thermo Scientific, USA). Mass Spectrometry experiments were performed on a Micro Mass Quadruple Time-of-Flight Ultima Global (Waters, Manchester, UK) and ion mobility mass spectrometry measurements were made on an in-house modified quadrupole time-of-flight mass spectrometer including a 5.1 cm drift cell^44^. Ions were produced by positive nano-electrospray ionisation (nESI), with a spray voltage range of 1.55–1.69 kV and a source temperature of 80 °C. nESI glass capillaries were prepared in-house from thin-walled borosilicate capillaries (Precision Instruments, Stevenage, UK) using a Flaming/Brown micropipette puller Model P-97 (Sutter Instrument company, Novato, CA, USA). The IM-MS drift cell was filled with Helium gas, to pressure of 3.56–3.75 Torr, measured using a baratron (MKS Instruments). The electric potential of the drift cell was varied from 12 to 2 Vcm^−1^. Ion arrival time distributions (ATDs) were recorded by synchronisation of the release of ions into the drift cell with mass spectral acquisition. ATDs were converted into collision cross section distributions using equation:


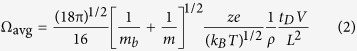


where Ω is the rotationally averaged collision cross section (Å^2^), m and m_b_ are the masses of the ion and the buffer gas, *z* is the ion charge, *e* is the elementary charge, k_B_ is the Boltzmann constant, ρ is the buffer gas density, T is the gas temperature, L is the drift cell length, V is the voltage applied across the drift cell (here 35 V) and t_D_ is the drift time. The arrival time of the ions (t_a_) includes the time spent within the mass spectrometer by outside of the drift cell, also known as the dead time (t_0_). The value for t_0_ is calculated by taking an average value of the intercept from a linear plot of average arrival time versus pressure/temperature and was subtracted from the arrival time to calculate drift time (t_D_):


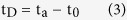


All processing was carried out using Mass Lynx V4.1 (Waters Corporation) and Origin 9.0 (Origin Lab Corporation, USA) software. Intensities of the CCSD peaks was directly taken from peak intensity during IM-MS experiments for three analytical repeats, in Mass Lynx V4.1 software (Waters Corporation).

## Additional Information

**How to cite this article**: Hagiwara, Y. *et al*. Consequences of point mutations in melanoma-associated antigen 4 (MAGE-A4) protein: Insights from structural and biophysical studies. *Sci. Rep.* **6**, 25182; doi: 10.1038/srep25182 (2016).

## Supplementary Material

Supplementary Information
