# Supplementary material for: Consequences of point mutations in melanoma-associated antigen 4 (MAGE-A4) protein: Insights from structural and biophysical studies
Source: Sci Rep. 2016 Apr 28;6:25182. doi: 10.1038/srep25182 (PMC4848555; doi:10.1038/srep25182)
Supplement: Supplementary Information [file srep25182-s1.pdf]

Supplementary Information for

Consequences of point mutations in melanoma-associated antigen 4 (MAGE-A4) protein: Insights from structural and biophysical studies

Yoshio Hagiwara[1], Lina Sieverling[1], Farina Hanif[1], Jensy Anton[1], Eleanor R Dickinson[2], Tam T.T. Bui[3], Antonina Andreeva[4], Perdita E Barran[2] Ernesto Cota[5] & Penka V. Nikolova[1]*

[1]King's College London, Faculty of Life Sciences & Medicine, Institute of Pharmaceutical Science, Franklin-Wilkins Building, 150 Stamford St, London, SE1 9NH, UK; [2]Michael Barber Centre for Collaborative Mass Spectrometry, Manchester Institute of Biotechnology, The University of Manchester, 131 Princess Street, Manchester, M1 7DN, UK; [3]Biomolecular Spectroscopy Centre, King's College London, The Wolfson Wing, Hodgkin Building, London SE1 1UL; [4]MRC-LMB, Francis Crick Avenue, Cambridge, CB2 0QH; [5]Imperial College London, Faculty of Natural Sciences, London, SW7 2AZ

*Correspondence should be addressed to:
Dr. Penka V. Nikolova
King's College London, Faculty of Life Sciences & Medicine, Institute of Pharmaceutical Science, Franklin-Wilkins Building, 150 Stamford St, London, SE1 9NH
E-mail: penka.nikolova@kcl.ac.uk

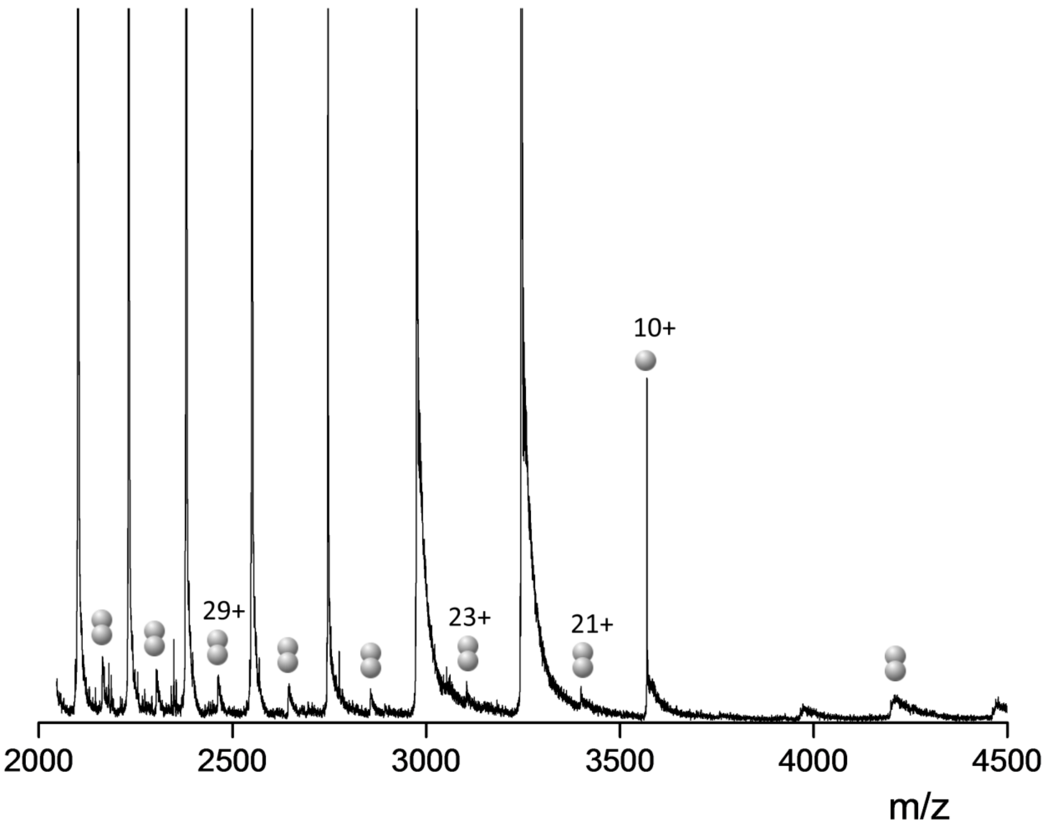

Supplementary Figure S1 nESI spectra adapted from Figure 7, showing a zoomed in section of 20 μM WT MAGE-A4 sprayed from buffered conditions 50 mM ammonium acetate. Low intensity unique m/z dimeric species are denoted by double spheres. The $[M+10H]^{10+}$ monomeric species is labelled for intensity comparison.

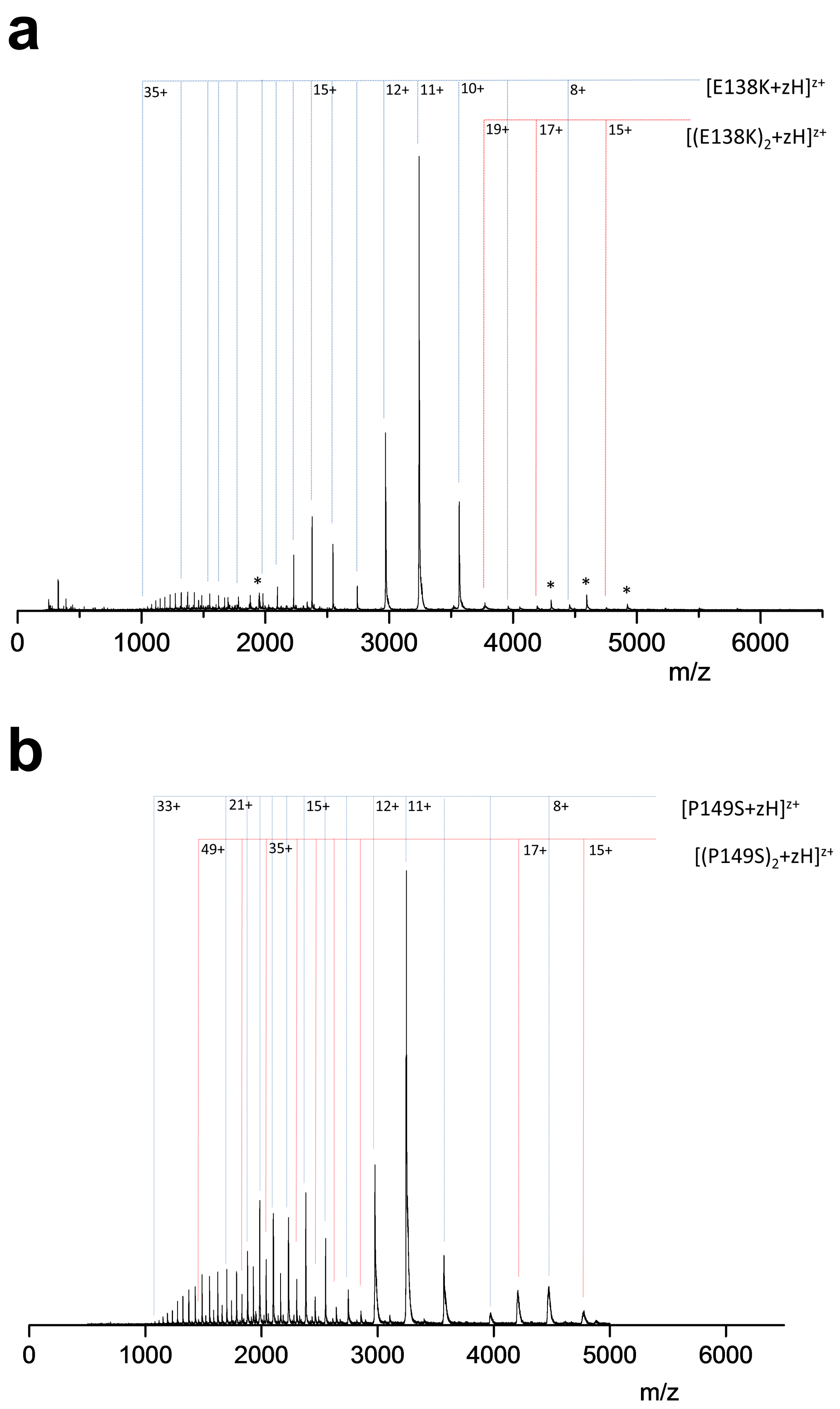
a
35+
15+
12+
11+
10+
8+
[E138K+zH]$^{z+}$
19+
17+
15+
[(E138K)$_2$+zH]$^{z+}$
*
*
*
*
0
1000
2000
3000
4000
5000
6000
m/z
b
33+
21+
15+
12+
11+
8+
[P149S+zH]$^{z+}$
49+
35+
17+
15+
[(P149S)$_2$+zH]$^{z+}$
0
1000
2000
3000
4000
5000
6000
m/z

c

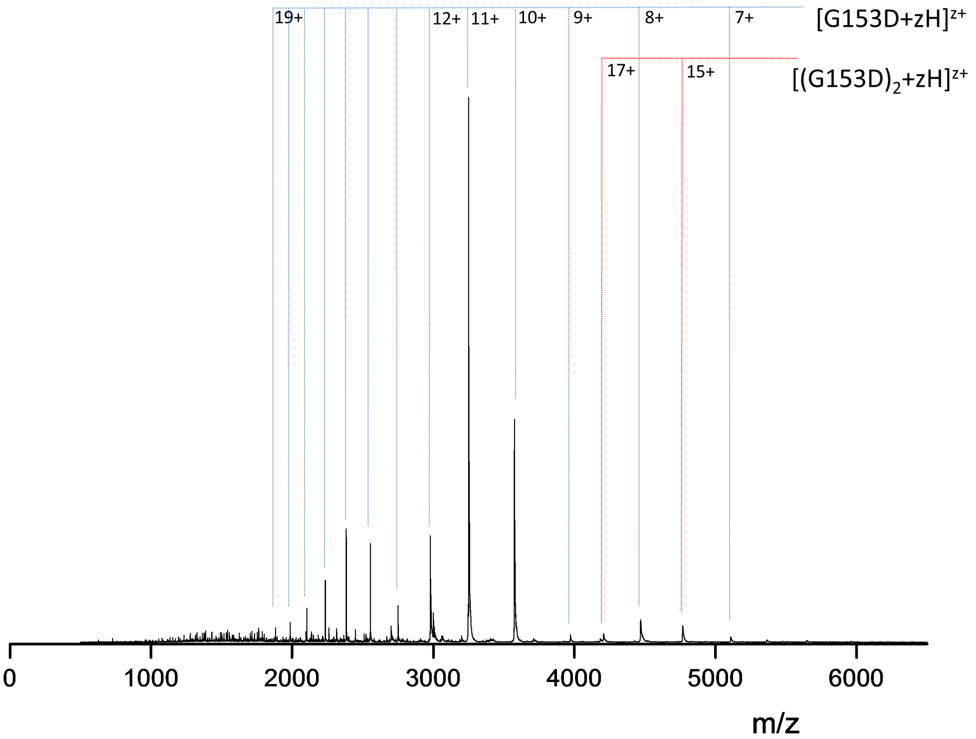
19+
12+
11+
10+
9+
8+
7+
[G153D+zH]z+
17+
15+
[(G153D)2+zH]z+
0
1000
2000
3000
4000
5000
6000
m/z

d

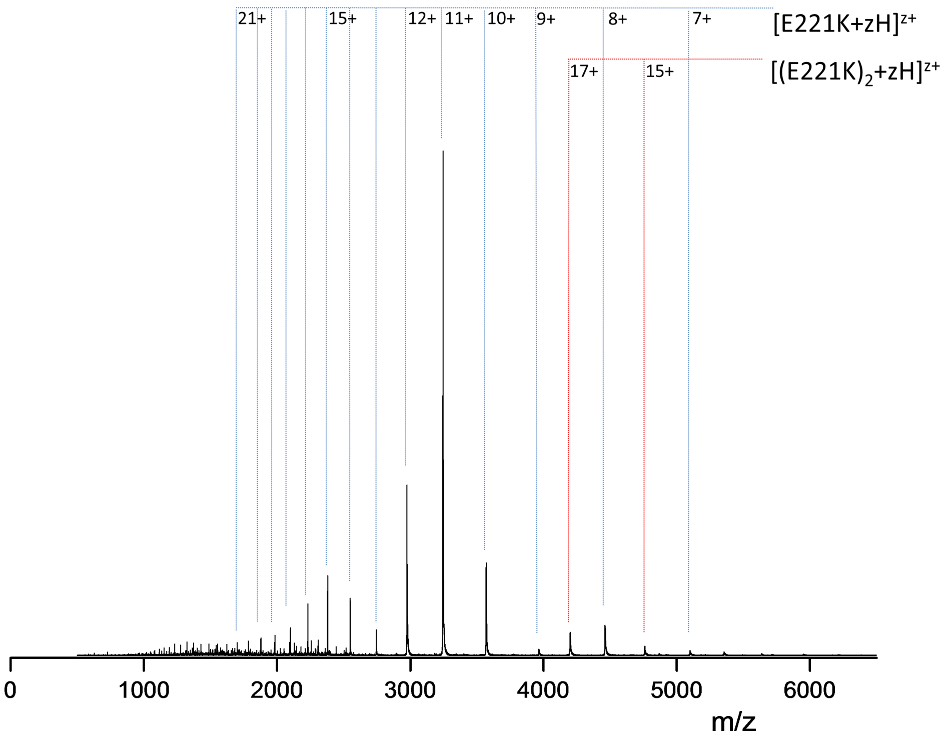
21+
15+
12+
11+
10+
9+
8+
7+
[E221K+zH]z+
17+
15+
[(E221K)2+zH]z+
0
1000
2000
3000
4000
5000
6000
m/z

e

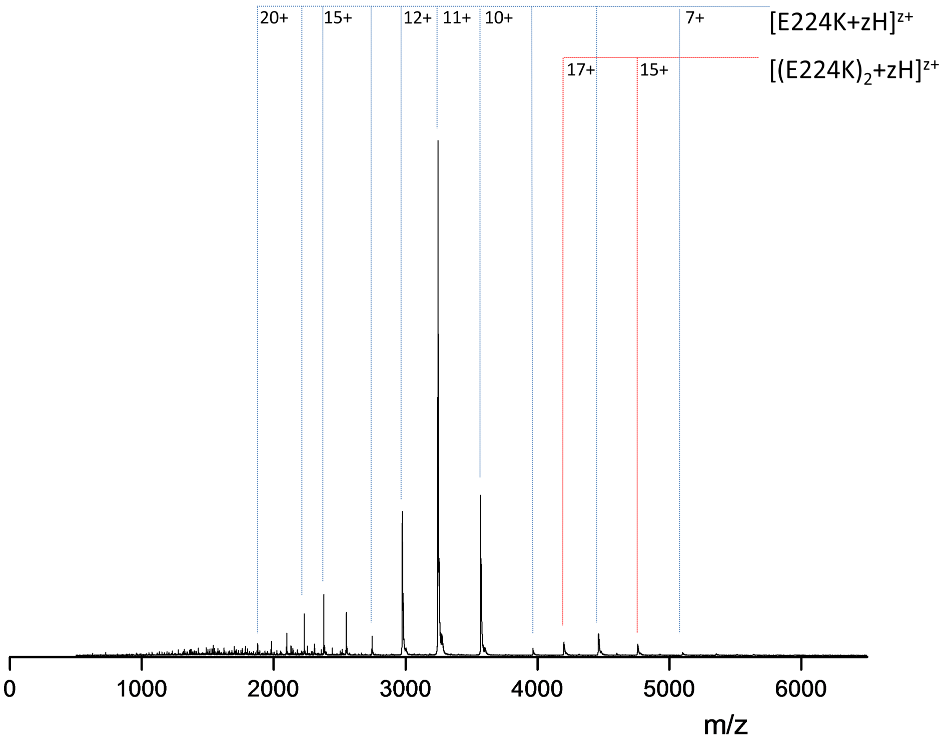
20+
15+
12+
11+
10+
7+
[E224K+zH]z+
17+
15+
[(E224K)2+zH]z+
0
1000
2000
3000
4000
5000
6000
m/z

f

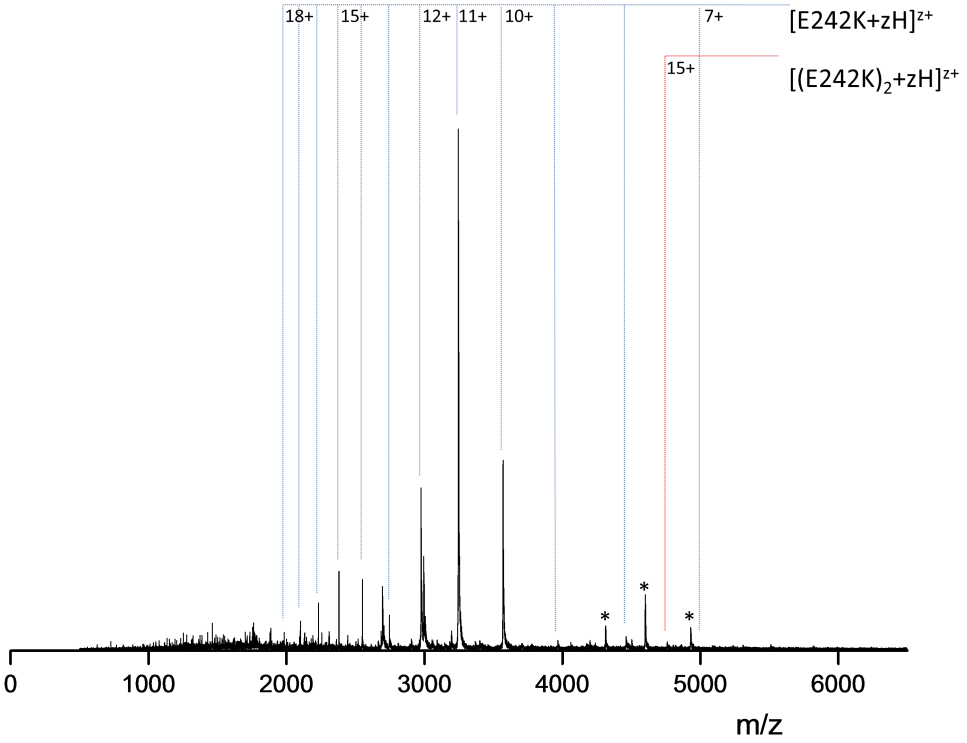
18+
15+
12+
11+
10+
7+
[E242K+zH]z+
15+
[(E242K)2+zH]z+
*
*
*
0
1000
2000
3000
4000
5000
6000
m/z

g

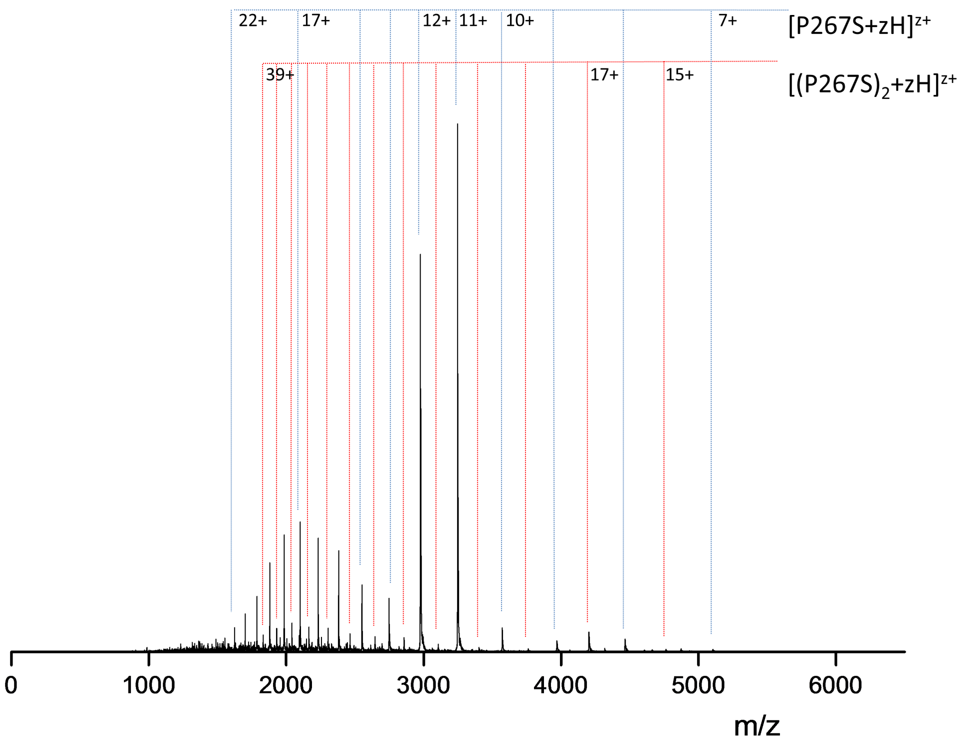
22+
17+
12+
11+
10+
7+
[P267S+zH]$^{z+}$
39+
17+
15+
[(P267S)$_2$+zH]$^{z+}$
0
1000
2000
3000
4000
5000
6000
m/z

h

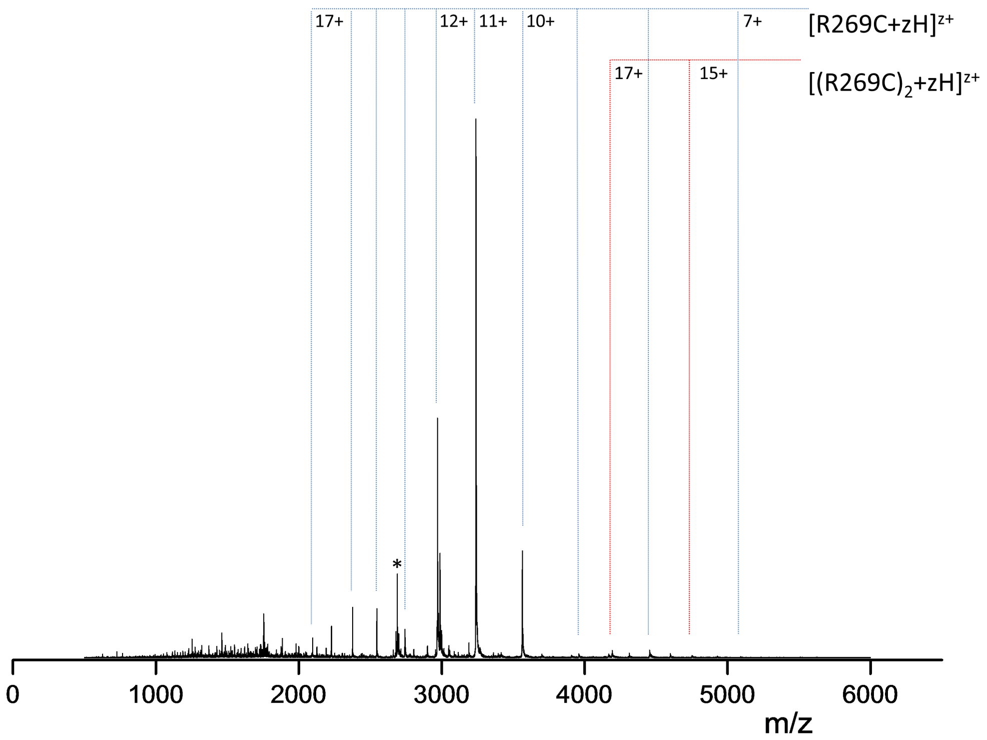
17+
12+
11+
10+
7+
[R269C+zH]$^{z+}$
17+
15+
[(R269C)$_2$+zH]$^{z+}$
*
0
1000
2000
3000
4000
5000
6000
m/z

i

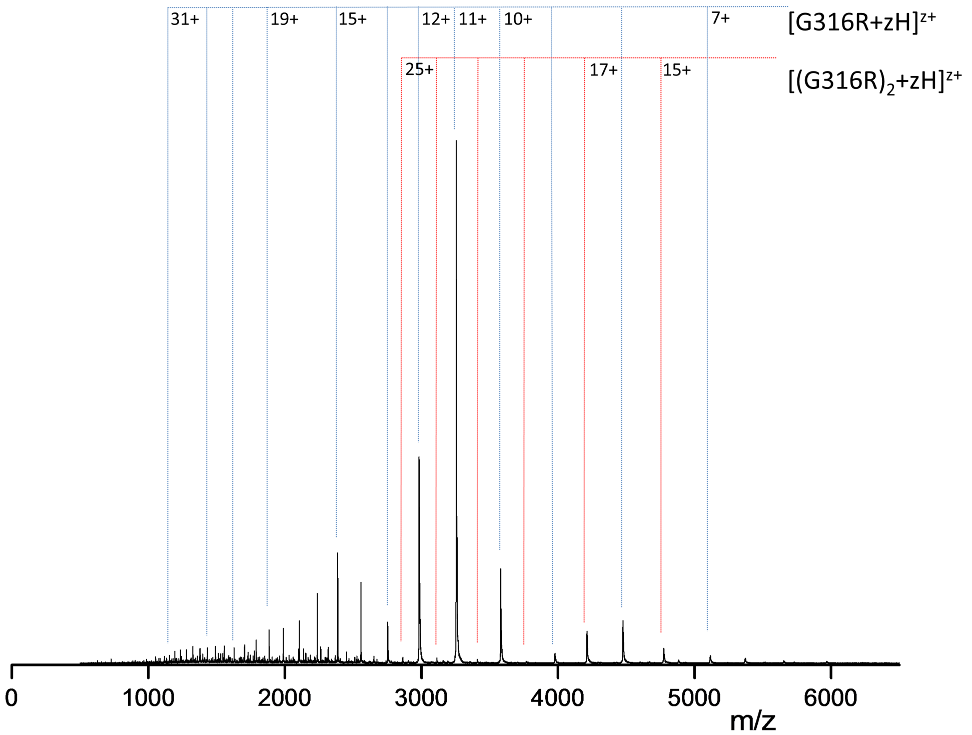

Supplementary Figure S2 nESI mass spectra of 20 µM MAGE-A4 mutants sprayed from buffered conditions of 50 mM Ammonium Acetate pH 6.8. Monomeric and dimeric species are depicted by blue and red dotted lines, respectively. Asterisks denote contaminant peaks in the spectra. The tested mutants are (**a**) E138K, (**b**) P149S, (**c**) G153D, (**d**) E221K, (**e**) E224K, (**f**) E242K, (**g**) P267S, (**h**) R269C and (**i**) G316R.

a

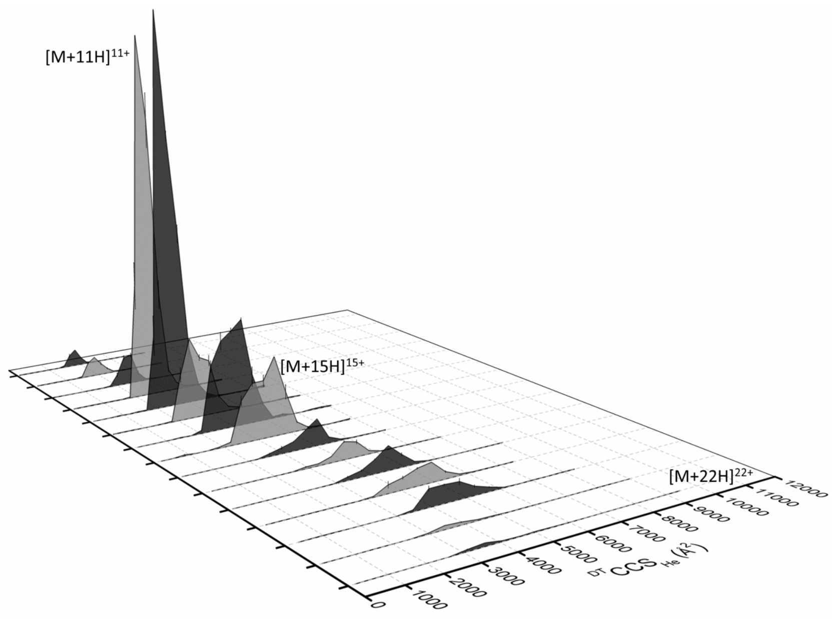

b

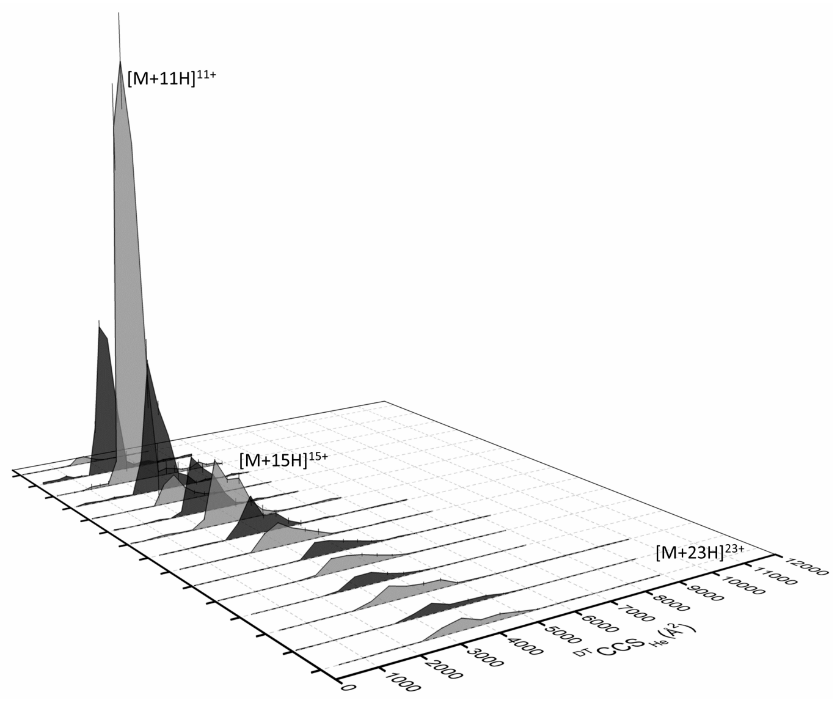

Supplementary Figure S3 Collison cross section distributions (CCSDs) derived from arrival time distributions (ATDs) for (**a**) E221K MAGE-A4 and (**b**) E242K MAGE-A4 at drift voltage 35 V. The x-, y- and z-axes represent collision cross section (CCS, Å^2), intensity directly acquired from IM-MS experiment, and charge state, respectively. Significant charge states have been labelled.

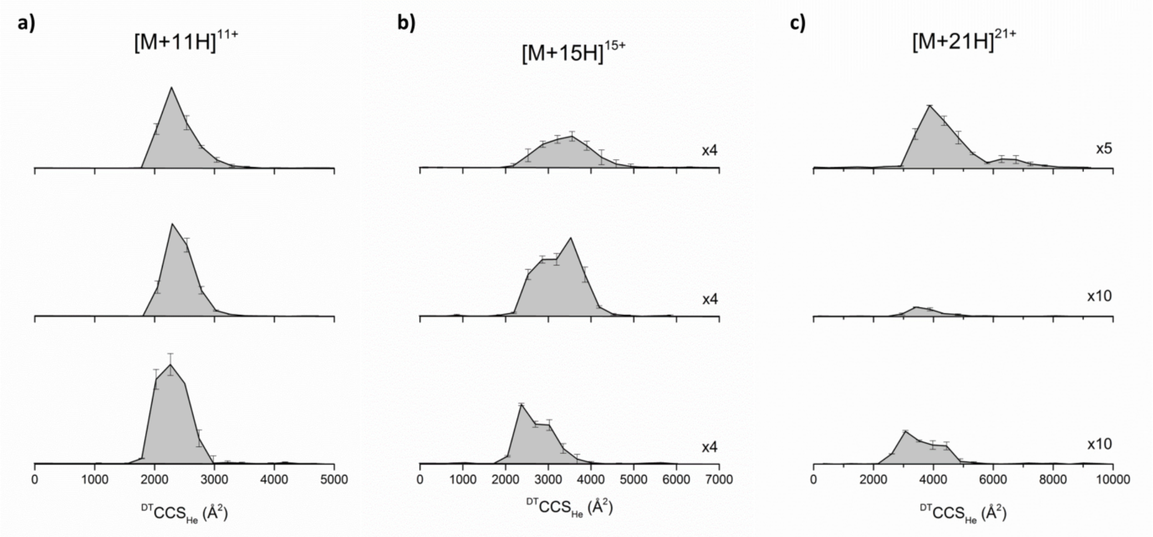

Supplementary Figure S4 Stacked collision cross section distributions (CCSDs) derived from arrival time distributions (ATDs) for wild-type MAGE-A4 (top panel), E221K (middle panel) and E242K (bottom panels) for (**a**) $[M+11H]^{11+}$, (**b**) $[M+15H]^{15+}$ and (**c**) $[M+21H]^{21+}$ at drift voltage 35 V. The x-, y- and z-axes represent collision cross section (CCS, $Å^2$), intensity directly acquired from IM-MS experiment, and charge state, respectively. Magnification of some CCSDs for visibility is noted.
